# *Fusobacterium nucleatum* bacteremia complicated with intracranial *Porphyromonas gingivalis* and *HSV-1* infection: a case report and literature review

**DOI:** 10.1186/s12879-024-09078-6

**Published:** 2024-02-20

**Authors:** Sumei Wang, Yaqiong Tian, Yujing Wu, Zhen Yu, Jinjuan Zhang, Jiandong Zhang, Shuye Liu

**Affiliations:** The Third Central Hospital of Tianjin, Tianjin Key Laboratory of Extracorporeal Life Support for Critical Diseases, Artificial Cell Engineering Technology Research Center, Tianjin Institute of Hepatobiliary Disease, Tianjin, China

**Keywords:** *Porphyromonas gingivalis*, *HSV-1*, *Fusobacterium nucleatum*, Infection

## Abstract

**Background:**

*Fusobacterium nucleatum* (*F. nucleatum*) belongs to the genus Fusobacterium, which is a gram-negative obligate anaerobic bacterium. Bacteremia associated with *F. nucleatum* is a serious complication, which is not common in clinic, especially when it is combined with other intracranial pathogenic microorganism infection. We reported for the first time a case of *F. nucleatum* bacteremia combined with intracranial *Porphyromonas gingivalis* (*P. gingivalis*) and *herpes simplex virus type 1*(*HSV-1*) infection.

**Case presentation:**

A 60-year-old woman was admitted to our hospital with a headache for a week that worsened for 2 days. Combined with history, physical signs and examination, it was characterized as ischemic cerebrovascular disease (ICVD). *F. nucleatum* was detected in blood by matrix-assisted laser desorption/ionization time-offight mass spectrometry (MALDI-TOF-MS). Meanwhile, *P. gingivalis* and *HSV-1* in cerebrospinal fluid (CSF) were identified by metagenome next generation sequencing (mNGS). After a quick diagnosis and a combination of antibiotics and antiviral treatment, the patient recovered and was discharged.

**Conclusion:**

To our knowledge, this is the first report of intracranial *P. gingivalis* and *HSV-1* infection combined with *F. nucleatum* bacteremia.

## Introduction

*Fusobacterium nucleatum* (*F. nucleatum*) is a gram-negative non-sporium obligate anaerobic bacterium, which can cause severe microbial infections in a variety of organs, often accompanied by serious sequelae. It is most commonly found in the mouth, and can also be distributed in the head and neck, lung and pleura, gastrointestinal organs and female reproductive tract [1]. Although *F. nucleatum* can cause serious infection, it is uncommon in clinical practice, accounting for less than 1% of the total number of anaerobic bacteria [2]. This may be due to the limited sensitivity and specificity of traditional culture-based pathogen diagnosis methods, which may underestimate the prevalence of infections caused by anaerobic bacteria until new laboratory testing techniques are available [3]. We report a rare case of *F. nucleatum* bacteremia combined with intracranial *P. gingivalis* and *HSV-1* infection.

## Case presentation

A 60-year-old female patient was admitted to the hospital with a one-week headache that worsened for two days. She had a past medical history of hypertension and type 2 diabetes mellitus, and no other abnormalities were found during physical examination. Brain CT showed bilateral ischemic foci in the basal ganglia region. After admission, the patient was given clopidogrel bisulfate tablets for antiplatelet aggregation, atorvastatin calcium tablets for lipid regulation and plaque stabilization, butylphthalide sodium chloride injection to improve cerebral metabolism, kerlikon to promote the establishment of collateral circulation, neurontopin for neurological nourishment, pantoprazole for prevention of stress ulcers, and monitoring of blood pressure and blood glucose, as well as symptomatic and supportive treatment.

The patient had a sudden onset of chills and high fever with a temperature of 39.8 °C after 3 days. Laboratory studies were as follows: white blood cell count 6.55 × 10^9^ /L with 81.7% neutrophils, red blood cell count 3.94 × 10^12^/L, hemoglobin 126 g/L, platelet count 207 × 10^9^ /L, Procalcitonin > 2ng/mL, C-reactive protein 394 µg/mL, CSF red blood cell count 20/µL, CSF white blood cell count 150/µL (neutrophils, 70%; lymphocytes, 30%), CSF glucose at 5.93mmol/L, chloride 120.6mmol/L, and protein at 54.51 mg/dL. CSF cryptococcus, acid-fast staining, and gram staining were all negative. Brain magnetic resonance imaging (MRI) showed multiple ischemic lesions in the bilateral basal ganglia, bilateral central half oval area, bilateral frontoparietal area, and left temporal occipital junction area. Ceftriaxone and ganciclovir were given empirically for anti-infective treatment. After 4 days of empirical anti-infective treatment with ceftriaxone and ganciclovir, the patient still had a low-grade fever. To further clarify the infection, blood was drawn for culture and lumbar puncture was performed for CSF metagenome next generation sequencing (mNGS). Nucleic acid extraction was performed using the Universal DNA/RNA Extraction Kit and quality monitoring was performed using Qubit 4.0. Library construction was performed using the KS619-DNAmN48 Pathogenic Microorganisms Metagenomic DNA Detection Kit (Reversible End-Termination Sequencing Method). The monitored libraries were up-sequenced using an Illumina NextSeq550 sequencer and compared with the macro system for database comparison.

**Fig. 1:**
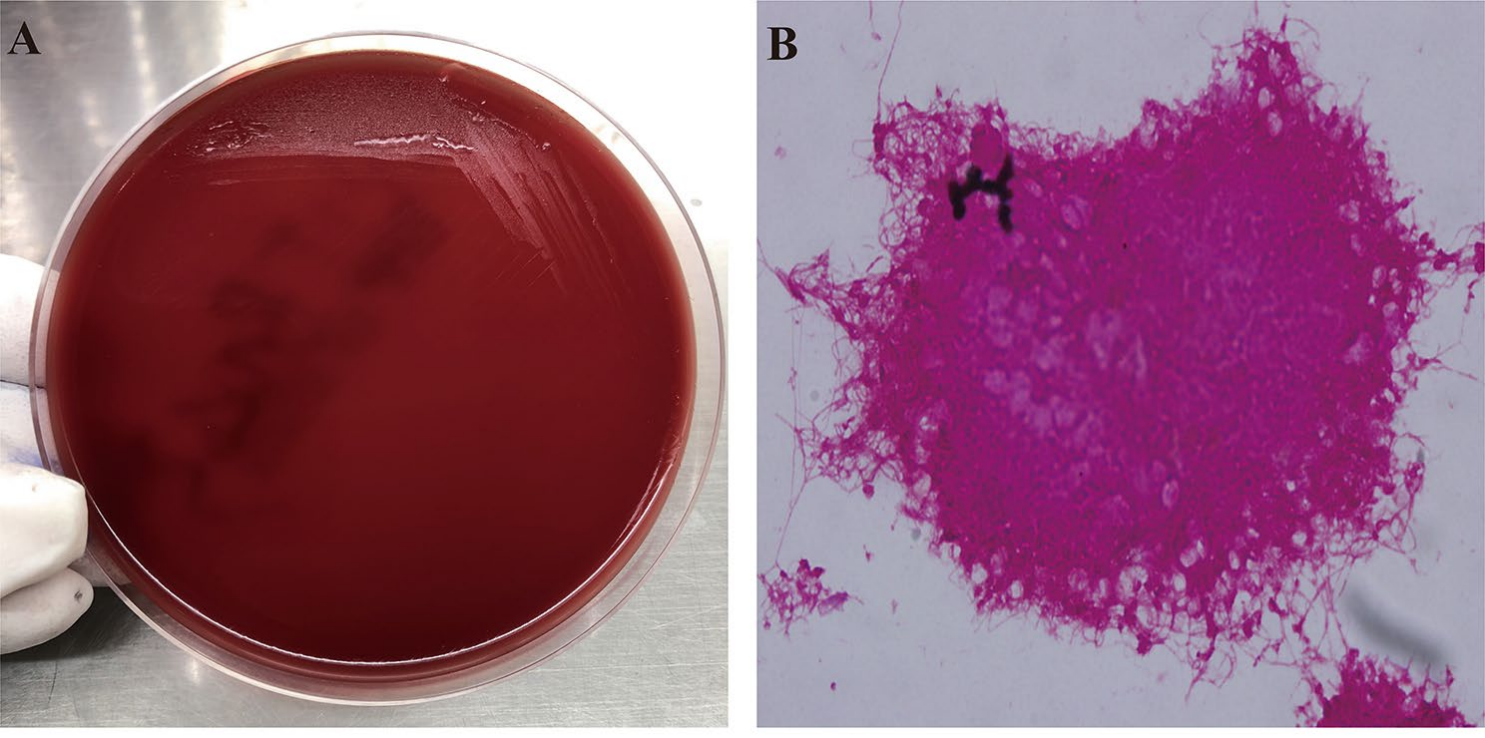
**A**: Growth of *F. nucleatum* on blood agar plates after 24 h of incubation. **B**: Gram-staining of *F. nucleatum*

Based on the sequencing results, *P. gingivalis* and *HSV-1* were detected, and *F. nucleatum* was detected in blood by matrix-assisted laser desorption/ionization time-of fight mass spectrometry (MALDI-TOF-MS) (Fig. 1). The anti-infective regimen was adjusted to ceftriaxone (2.0 iv Q12H) in combination with metronidazole (0.5 iv Q8H) and antiviral therapy with acyclovir (10 mg/kg iv Q8H), and the patient was free of significant headache and fever after 5 days. Re-examination of CSF and blood culture were negative, and a one-month telephone follow-up after discharge revealed no significant discomfort.

## Discussion and conclusions

*F. nucleatum* is an adherent anaerobic oral commensal involved in plaque formation and is associated with oral infectious diseases, gastrointestinal diseases (colorectal cancer, inflammatory bowel disease, and appendicitis), and a variety of other infections and abscesses [4]. Although we found 9 cases of adult *F. nucleatum* bloodstream infection on PubMed (Table 1). To our knowledge, this is the first case of *F. nucleatum* bacteremia combined with intracranial *P. gingivalis* and *HSV-1* infection.

**Table 1:** Summary of 9 case reports identified *F.nucleatum* bloodstream infection

Case	Country	Age(sex)	Medical history	WBC	CRP	Fever	Antibiotics	References
1	Japan	59(M)	hypertension	High	High	Present	- Imipenem/cilastatin 500 mg BD (4 days) - Oral clindamycin 600 mg BD (UTP)	Kajiya et al., 2008 [11]
2	Canada	24(M)	Healthy	High	N/A	Present	- IV metronidazole (4 weeks) - Oral metronidazole (2 weeks)	Cheung et al., 2007 [12]
3	France	68(M)	lung tuberculosis, genitourinary tract tuberculosis, thrombocytopenia, and pulmonary embolisms	Low	High	Present	- IV cefotaxime 1 g Q8H - IV metronidazole 500 mg Q8H (24 days) - Oral metronidazole (2 weeks) - Enoxaparin 20 mg daily (UTP)	Etienne et al., 2001 [13]
4	USA	76(F)	hypertension, chronic obstructive pulmonary disease, and tobacco use disorder	N/A	N/A	Present	- vancomycin 15 mg/kg Q12H) (4 days) - cefepime 2 g Q12H (4 days) - metronidazole 500 mg Q8H (4 days)	Swaminathan and Aguilar, 2020 [14]
5	Japan	28(M)	Healthy	Low	High	Present	- Ceftriaxone (6 days) - Meropenem (14 days)	Takano et al., 2015 [15]
6	USA	30(M)	Healthy	High	N/A	Present	- Piperacillin/tazobactam and metronidazole (UTP) - Oral levofloxacin and metronidazole (UTP)	Fatakhov et al., 2013 [16]
7	Japan	44(M)	Healthy	High	High	Present	- Cefotiam (UTP) - Doripenem (19 days) - Oral levofloxacin (UTP)	Iwasaki et al., 2012 [17]
8	USA	25(M)	Healthy	High	High	Present	- Piperacillin/tazobactam (UTP) - IV penicillin (6 weeks)	Handler et al., 2011 [18]
9	USA	44(M)	paranoid schizophrenia, smoking, gonorrhea and trichomoniasis	High	N/A	Present	- IV ampicillin/sulbactam and ciprofloxacin (5 days) - IV ampicillin/sulbactam and metronidazole (13 days) - IV ampicillin/sulbactam (15 days) - Oral amoxicillin/clavulanate (1 month)	Treszezamsky et al., 2010 [19]

Abbreviations: N/A, not available; UTP, Unspecified time period; IV, Intravenous; BD, twice daily.

The large number of adhesins on the surface of *F. nucleatum* recognizes various complementary structures on the surfaces of bacteria and host cells, which are important for colonization and establishment of infection in susceptible hosts [5].The most characteristic adhesin is RadD, which is an autonomous transporter protein of about 350 kDa in size that adheres to early-stage colonizing bacteria, late-stage pathogenic bacteria, and immune cells in the oral cavity. In addition, RadD mediates the binding of Clostridium nucleatum to other bacteria or fungi, such as *Streptococcus*, *Staphylococcus aureus* and *Candida albicans*. *F. nucleatum* naturally increases the pH of its local environment by consuming amino acids and releasing ammonia, which allows the growth of acid-sensitive bacteria such as *P. gingivalis*. At the same time, the mucopolysaccharide layer of the bacterial cell wall can induce abscess formation.

*F. nucleatum* elicits a variety of host responses. *F. nucleatum* is a potent stimulator of the inflammatory cytokines interleukin 6 (IL-6), IL-8, and tumor necrosis factor α (TNFα) [6]. Binding of *F. nucleatum* to NK cells activates the inflammatory response associated with periodontal disease. In colorectal cancer cells, *F. nucleatum* not only activates the inflammatory response, but also activates oncogene and Wnt gene expression, all of which are hallmarks of tumorigenesis [7]. *HSV-1* was found to be positively correlated with *F. nucleatum* in the site of peri implant inflammation. Our case shows that *F. nucleatum* bacteremia combined with intracranial *HSV-1* infection [8]. The first line of central nervous system immune defense against *HSV-1* comes from the recognition of pathogen-associated molecular patterns (PAMPs) by the Toll-like receptor (TLR) family. Response of TLR2 or TLR9 to neuroglial cells leads to the production of type I interferon (IFN), IL-15, TNF, and chemokine CCL2, recruitment of macrophages. Whether there is a potential relationship of immune response between *F. nucleatum* and *HSV-1* needs to be further investigated [9].

Lemierre syndrome is a rare upper airway infection with life-threatening secondary septic thrombophlebitis of the internal jugular vein or external jugular vein, usually seen in previously healthy young adults, characterized by bacteremia, thrombophlebitis of the internal jugular vein, and metastatic septic embolus secondary to acute pharyngeal infection. The most common pathogen is *F. necrophorum*, followed by *F. nucleatum* and anaerobes such as *streptococcus*, *staphylococcus* and *klebsiella pneumoniae*. The pathogenic foci originate primarily from pharyngitis or tonsillitis [10].

For infections caused by *F. nucleatum*, treatment generally includes anti-infective therapy, abscess drainage and surgical debridement. *Fusobacterium* is sensitive to many commonly used clinical antibiotics, but the sensitivity rate tends to decrease, or may be resistant to vancomycin, erythromycin, amoxicillin, ampicillin, tetracycline and so on. Some strains can produce β-lactamase, and drugs that incorporate β-lactamase inhibitors (e.g., clavulanic acid) may be used for treatment. Drugs suitable for empirical treatment are metronidazole, clindamycin and amoxicillin-clavulanate potassium, and other effective antimicrobials include carbapenems and chloramphenicol. However, it should be noted that most of these infections are mixed flora infections, including microaerobic and aerobic streptococci, and combinations of drugs need to be considered to cover all pathogens. In-hospital bacteremia is an important predictor of mortality, independent of other parameters of disease severity. *F. nucleatum* bacteremia is associated with invasive and lethal infections, especially pneumonia and abdominal infections, and early recognition of such serious infections with appropriate antibiotic therapy and surgical debridement or drainage is important. Also, by investigating the epidemiologic background of *F. nucleatum* infections, we can effectively reveal the high-risk groups for various related diseases and facilitate early detection and prevention in the population.

In conclusion, we found that prompt recognition of *F. nucleatum* infection and the presence or absence of co-infection with other microorganisms is important for the treatment and prognosis of the patient.

## Data Availability

No datasets were generated or analysed during the current study.

## Abbreviations

*F. nucleatum*: *Fusobacterium nucleatum*
*P. gingivalis*: *Porphyromonas gingivalis*
*HSV-1*: *Herpes simplex virus type 1*
ICVD: Ischemic cerebrovascular disease
MALDI-TOF-MS: Matrix-assisted laser desorption/ionization time-offight mass spectrometry
CSF: Cerebrospinal fluid
mNGS: Metagenome next generation sequencing
MRI: Magnetic resonance imaging
